# Causes of Death and Prognostic Factors in Patients with Superficial Esophageal Cancer Curatively Treated by Endoscopic Therapy: A Multicenter Cohort Study

**DOI:** 10.3390/cancers18030445

**Published:** 2026-01-29

**Authors:** Koichiro Kawaguchi, Atsushi Yanagitani, Naoya Noguchi, Ryohei Ogihara, Kazuo Yashima, Hajime Isomoto

**Affiliations:** 1 Division of Gastroenterology and Nephrology, Faculty of Medicine, Tottori University, Yonago 683-8504, Japan; koichiro@tottori-u.ac.jp (K.K.); ryoheiogihara@gmail.com (R.O.); yashima@tottori-u.ac.jp (K.Y.); 2 Tottori Prefectural Central Hospital, Tottori 680-0901, Japan; yanagitani-a@tp-ch.jp; 3 Tottori Prefectural Kousei Hospital, Tottori 682-0804, Japan; haru-8677@outlook.jp

**Keywords:** superficial esophageal cancer, endoscopic resection, prognosis, cause of death, body mass index, survivorship

## Abstract

**Background**: Endoscopic resection has become a standard curative treatment for superficial esophageal cancer. However, limited data are available regarding long-term outcomes and causes of death after curative endoscopic treatment, particularly deaths unrelated to the primary esophageal cancer. This study aimed to clarify post-treatment prognosis and to identify factors associated with mortality in patients with superficial esophageal cancer who underwent curative endoscopic resection. **Methods**: We conducted a multicenter cohort study in Japan to evaluate survival outcomes, causes of death, and prognostic factors in patients with superficial esophageal cancer who achieved curative resection by endoscopic treatment. Patients were stratified according to endoscopic treatment indication categories (absolute, relative, and beyond indication). Five-year overall survival and disease-specific survival were analyzed, along with risk factors for all-cause and cause-specific mortality. **Results**: No disease-specific deaths were observed in patients with absolute or relative indication lesions, whereas the disease-specific 5-year survival rate was 81% in patients with beyond-indication lesions. When overall mortality, including deaths from other diseases and other malignancies, was evaluated, the 5-year survival rates were 92% for absolute indication cases, 85% for relative indication cases, and 69% for beyond-indication cases. Multivariate analysis identified low body mass index (BMI) and advanced age as independent risk factors for mortality. Notably, low BMI was significantly associated with non-cancer-related death. **Conclusions**: Even among patients who achieve curative endoscopic treatment for superficial esophageal cancer, long-term survival is limited by deaths from other malignancies and non-cancer-related causes. Low BMI represents a clinically important prognostic factor, underscoring the need for comprehensive post-treatment surveillance and supportive care beyond cancer control.

## Simple Summary

Endoscopic resection is an established curative treatment for superficial esophageal cancer and provides excellent disease-specific survival. However, in clinical practice, we have often experienced that patients who undergo curative endoscopic resection are subsequently lost due to causes unrelated to esophageal cancer. This clinical observation prompted us to investigate factors affecting long-term outcomes beyond cancer control. In this study, we evaluated the impact of body mass index (BMI) on survival after curative endoscopic resection. Although disease-specific survival was excellent across all BMI categories, a lower BMI was significantly associated with poorer overall survival, primarily due to increased non-cancer-related mortality. These findings highlight the importance of long-term surveillance and comprehensive patient management, particularly for underweight patients, even after curative endoscopic treatment.

## 1. Introduction

Esophageal cancer remains a major global health burden. According to the Global Burden of Disease (GBD) 2021 study, it ranks as the sixth leading cause of cancer-related mortality and the seventh most common cancer by global incidence [1]. In Japan, it ranks as the seventh most common cancer among men (2020) and the eighth leading cause of cancer-related mortality (2023), according to the National Cancer Center Japan (Cancer Information Service). More than 90% of esophageal cancers are squamous cell carcinomas, with alcohol consumption and smoking being well-established major risk factors.

According to the Japanese Classification of Esophageal Cancer, tumors invading up to the submucosa (SM) are classified as superficial cancer, while those confined to the mucosa (M) are defined as early cancer [2,3]. However, esophageal cancer is prone to early lymph node metastasis, and current clinical guidelines recommend endoscopic treatment primarily for early-stage disease [4]. Tumors invading the muscularis mucosae (MM, M3) or showing minimal submucosal invasion (<200 μm; SM1) reportedly carry a lymph node metastasis risk exceeding 10% [5] and are therefore classified as relative indications for endoscopic treatment under the Japanese guidelines. Importantly, in the Japanese guidelines, MM and SM1 cancers without lymphovascular invasion are still regarded as curatively resected by endoscopic treatment.

Esophagectomy remains a highly invasive procedure, with surgery-related mortality rates reported at approximately 1–2% nationwide, despite institutional variability. Consequently, early detection of lesions amenable to endoscopic treatment is critically important.

Recent advances in image-enhanced endoscopy (IEE) and magnifying endoscopy have increased the detection of early-stage esophageal cancer [6], and diagnostic systems for assessing invasion depth have become increasingly established [7], improving preoperative diagnostic accuracy. Current guidelines regard magnifying endoscopy combined with IEE as the most useful modality for depth assessment [4]. Nevertheless, precise preoperative evaluation of invasion depth and lympho-vascular involvement remains challenging, particularly in lesions suspected to be relative indication cases. Therefore, the ESD/EMR guidelines recommend diagnostic endoscopic resection for clinical MM/SM1 cancers, followed by pathological evaluation to determine curability and the need for additional treatment [4].

The development of endoscopic techniques, especially endoscopic submucosal dissection (ESD), has enabled en bloc resection even for extensive lesions, increasing the number of lesions curable by endoscopic therapy. Although post-ESD esophageal stricture was previously a major concern, a randomized clinical trial (JCOG1217) demonstrated the efficacy and safety of local steroid injection compared with oral steroid therapy, establishing local injection as the standard preventive treatment [8]. Furthermore, the efficacy and safety of additional chemoradiotherapy (CRT) for MM cancers with lympho-vascular invasion or SM cancers after endoscopic treatment were confirmed in JCOG0508, with surgical outcomes as a comparator [9].

As a result, endoscopic treatment is increasingly applied not only to relative indication lesions but also to clinical SM cancers as a “total biopsy” approach, further expanding the indications for endoscopic therapy. Furthermore, when the postoperative histopathological findings demonstrate that tumor invasion is confined to the mucosa or to superficial submucosa (SM1) and no lympho-vascular invasion is present, the resection is regarded as “curative resection [2,3,4].”

Esophageal cancer survivors are at increased risk of developing second primary malignancies, even after definitive treatment. In a large cohort analysis, approximately 14% of patients developed a second primary cancer, with the most common sites being the head and neck, lung, stomach, and colorectal regions [10]. Although head and neck cancer is the most frequently reported second primary cancer internationally [11], recent Japanese studies have shown that gastric cancer remains a common synchronous or metachronous malignancy in patients with esophageal squamous cell carcinoma even in the era of declining *Helicobacter pylori* infection rates [12,13]. These findings highlight the importance of comprehensive post-treatment surveillance beyond the esophagus, particularly after curative endoscopic resection. Although early-stage detection of such malignancies allows curative treatment, deaths due to other malignancies or non-cancer causes are frequently encountered during post-treatment surveillance. Despite extensive data on stage-specific survival of esophageal cancer, few studies have addressed the ultimate causes of death following curative endoscopic treatment [14].

Therefore, this study aimed to clarify real-world outcomes after curative endoscopic treatment for superficial esophageal cancer, focusing particularly on causes of death other than esophageal cancer and factors associated with poor prognosis.

## 2. Materials and Methods

### 2.1. Study Design and Patients

Patients with superficial esophageal cancer who underwent endoscopic resection were retrospectively and prospectively enrolled from three tertiary hospitals located in eastern, central, and western Tottori Prefecture: Tottori Prefectural Central Hospital, Tottori Prefectural Kousei Hospital, and Tottori University Hospital. Based on the final pathological assessment of the resected specimen, patients were categorized into two groups: a curative resection group and a non-curative (beyond-indication) resection group. The primary focus of the analysis was on survival outcomes in patients who achieved curative resection, with additional comparative analyses performed for patients who did not meet the criteria for curative resection.

Curative endoscopic treatment was defined according to the Japanese Classification of Esophageal Cancer [1,2]. Briefly, cancers confined to the intraepithelial layer or lamina propria mucosae (M1/M2) without lymphovascular invasion were regarded as absolute indication lesions, and tumors invading the muscularis mucosae or superficial submucosa (MM/SM1 < 200 μm) without lymphovascular invasion were regarded as relative indication lesions. Lesions fulfilling these criteria were considered to have achieved curative resection. In contrast, tumors with lymphovascular invasion or submucosal invasion ≥ SM2 were classified as beyond-indication lesions and defined as non-curative resection. For Barrett’s adenocarcinoma, lesions without lymphovascular invasion with invasion up to the deep muscularis mucosae of the duplicated muscularis mucosae were included within the curative category [2,3,4].

Given the difficulty of accurately diagnosing invasion depth and lymphovascular involvement before endoscopic resection, particularly in clinical M3/SM1 lesions, endoscopic submucosal dissection is often performed in a diagnostic setting (“diagnostic ESD”), and additional therapy such as chemoradiotherapy or surgery is considered according to the final pathological results. In keeping with this clinical practice, outcomes and causes of death were analyzed not only in the curative resection group but also in patients with beyond-indication lesions confined to the submucosa (T1b superficial cancer).

This study was supported by a Japanese government research grant and included ambispective cohorts: a retrospective cohort from 2008 to 2017 and a prospective cohort extending to 2024. Esophageal cancer frequently presents with multiple lesions, either synchronous or metachronous, and this pattern was also observed in the present study cohort. For patients with multiple lesions, the index lesion was defined as follows: lesions treated within one year were regarded as synchronous lesions, and the lesion with the greater depth of invasion was designated as the index lesion. In cases where multiple lesions were treated more than one year apart, the lesion that was treated first was defined as the index lesion. The date of endoscopic treatment for the index lesion was defined as time zero for all survival analyses.

### 2.2. Follow-Up and Outcome Ascertainment

Follow-up data were collected in April 2025. Prognostic information was obtained using a multi-step approach:

(1) Detailed review of electronic medical records at the treating institutions;
(2) When patients were followed at other hospitals, written requests for clinical information from the receiving institutions;
(3) Direct telephone interviews with patients or family members when necessary; and
(4) For cases in which survival status could not be confirmed by these methods, vital status and cause of death were verified using the Japanese National Cancer Registry and the resident registry (family register).

The cause of death was determined based on the most reliable available source among medical records, referral hospital reports, family interviews, and registry data. Discrepancies were resolved by consensus review among the investigators.

Causes of death were classified into three categories: death due to esophageal cancer, death due to other malignancies, and death due to non-malignant diseases.

### 2.3. Endpoints

The primary endpoints were the 5-year survival rates, causes of death, and mortality risk factors in patients who achieved curative resection after endoscopic treatment.

The secondary endpoints were comparisons of 5-year survival rates and causes of death according to endoscopic treatment indication categories, including absolute, relative, and beyond-indication lesions.

### 2.4. Statistical Analysis

All statistical analyses were conducted to evaluate long-term survival outcomes and prognostic factors in patients with superficial esophageal cancer who underwent endoscopic resection. Continuous variables are expressed as medians with interquartile ranges, and categorical variables as frequencies and proportions. Between-group comparisons were performed using the Mann–Whitney U test for continuous variables and the chi-square or Fisher’s exact test for categorical variables, as appropriate.

Overall survival (OS) was estimated using the Kaplan–Meier method and compared between groups using the log-rank test. Univariable and multivariable analyses for OS were performed using Cox’s proportional hazards regression models. Hazard ratios (HRs) with 95% confidence intervals (CIs) were calculated. Variables with *p* < 0.10 in univariable analyses and those considered clinically relevant were included in multivariable models.

For cause-specific mortality, competing risk analyses were performed. Cumulative incidence functions were estimated for each cause of death, and differences between groups were assessed using Gray’s test. In addition, the Fine–Gray subdistribution hazards model was used to evaluate risk factors for cause-specific death, treating deaths from other causes as competing events. Subdistribution hazard ratios (sHRs) with 95% CIs were calculated.

All covariates were defined at baseline (time zero, the date of endoscopic treatment for the index lesion). BMI was calculated from body weight and height at that time and was not treated as a time-dependent variable. Changes in BMI, recurrence, and additional treatments during follow-up were not modeled as time-varying covariates.

All statistical analyses were performed using IBM SPSS Statistics version 29 (IBM Corp., Armonk, NY, USA) and EZR version 1.54 (Saitama Medical Center, Jichi Medical University, Saitama, Japan). All tests were two-sided, and a *p* value < 0.05 was considered statistically significant.

## 3. Results

### 3.1. Patient Characteristics

**Figure 1:**
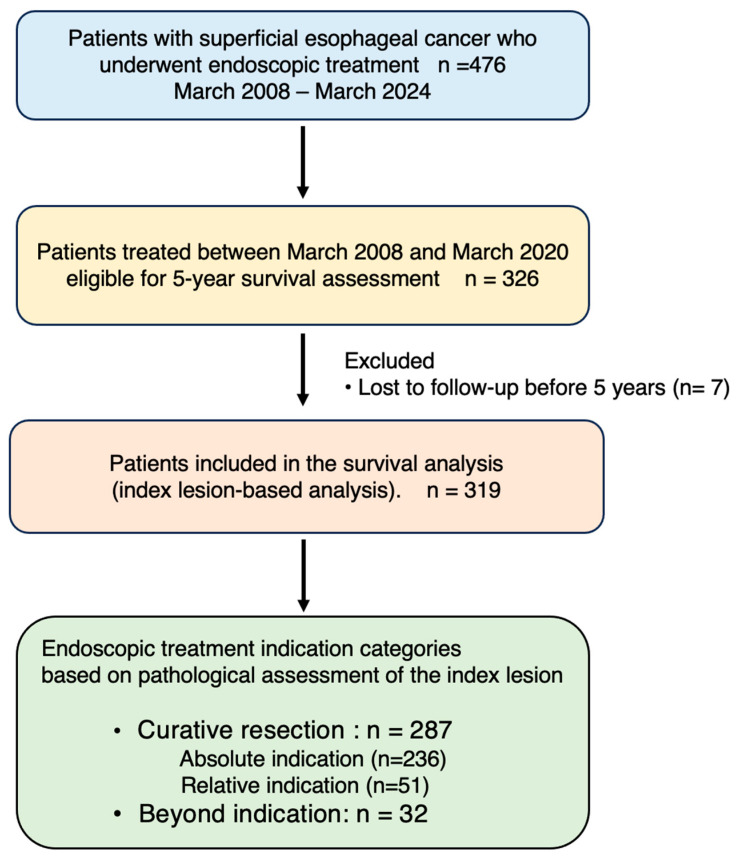
Study flow diagram.

From March 2008 to March 2024, 476 patients with superficial esophageal cancer underwent endoscopic treatment. Of these, 326 patients treated between March 2008 and March 2020 were eligible for assessment of 5-year survival outcomes, and 7 patients were excluded due to insufficient follow-up. Consequently, 319 patients were included in the final survival analysis. Figure 1 shows the flow diagram of patient selection and inclusion in the present study.

The mean and median patient ages were 69 and 70 years. There were 281 male patients and 38 female patients. Eighty-two lesions were synchronous or metachronous multiple lesions, and some patients had lesions spanning multiple indication categories. As described above, each patient’s prognosis was evaluated based on the index lesion, using the date of endoscopic treatment for the index lesion as the reference time point. The mean follow-up period for all 319 patients, including those with beyond-indication lesions, was 94 months (median, 89 months).

Among the 319 patients, 287 achieved curative resection, including 236 classified as absolute indication and 51 as relative indication, while the remaining 32 patients had beyond-indication lesions. Baseline patient characteristics according to endoscopic treatment indication are summarized in Table 1.

A total of 476 patients with superficial esophageal cancer underwent endoscopic treatment between March 2008 and March 2024. Among them, 326 patients treated between March 2008 and March 2020 were eligible for 5-year survival assessment. After excluding 7 patients lost to follow-up within 5 years, 319 patients were included in the survival analysis. Prognostic evaluation was performed on a patient basis using the index lesion, and patients were classified into absolute, relative, and beyond-indication groups according to pathological findings.

Curative resection was defined according to the Japanese Classification of Esophageal Cancer.

### 3.2. Treatment Outcomes and Prognosis

The en bloc complete resection rate achieved by ESD was high, and residual or local recurrence was rarely observed, even in extensive lesions. Positive or indeterminate lateral margins were observed in 10%, 14%, and 12% of absolute indication, relative indication, and beyond-indication lesions, respectively. However, curative outcomes were achieved in these cases through additional endoscopic treatments such as argon plasma coagulation or repeat ESD.

### 3.3. Analysis of Deaths

#### 3.3.1. Five-Year Survival and Cause of Death

Table 2 summarizes the causes of death according to curative and non-curative resection status.

Within five years, a total of 36 of the 319 patients died during follow-up.

In the curative resection group, no deaths were attributable to esophageal cancer, resulting in a 5-year cause-specific survival (CSS) rate of 100%. Among the 26 deaths observed in this group, causes of death were equally divided between other malignancies and non-cancer-related diseases.

In contrast, in the non-curative resection group, six patients died of esophageal cancer, yielding a 5-year CSS of 81%. In addition, two patients died of other malignancies and two died of non-cancer causes.

#### 3.3.2. Competing Risk Analysis of Cause-Specific Mortality

**Figure 2:**
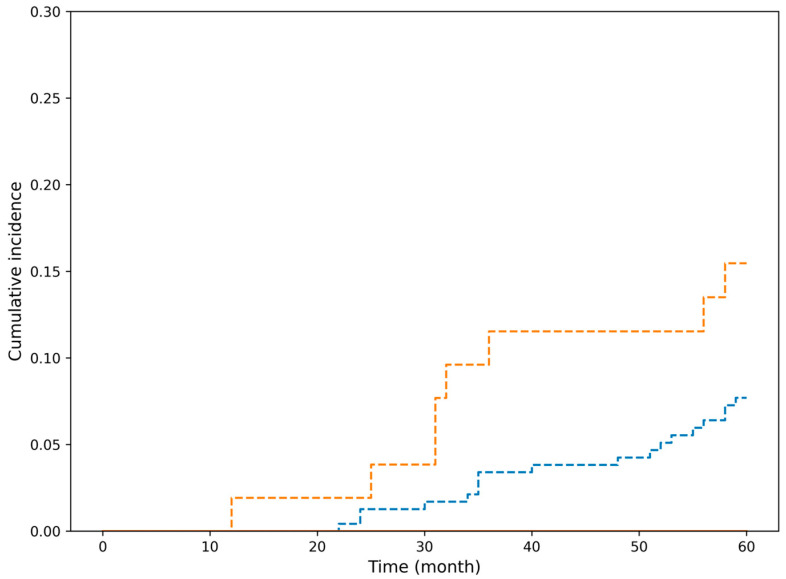
Cumulative incidence of esophageal cancer-specific mortality according to endoscopic resection indication status in the curative treatment cohort. Blue dashed line: The incidence of death from other malignancies and non-cancer causes in absolute indication patients. Orange dashed line: The incidence of death from other malignancies and non-cancer causes in relative indication patients. In both absolute and relative indication groups, the cumulative incidence of esophageal cancer-specific death remained 0% throughout the 5-year follow-up period, whereas the incidence of death from other malignancies and non-cancer causes increased steadily over time. Gray’s test was not performed for death from esophageal cancer because no such events occurred during the follow-up period.

Because no esophageal cancer-related deaths occurred in the curative resection group, competing risk methods were used to appropriately evaluate the cumulative incidence of non-esophageal cancer-related mortality. To account for competing risks between esophageal cancer-related death and death from other causes, cumulative incidence functions were estimated using the Fine–Gray subdistribution hazards model. Cumulative incidence functions are shown for death from esophageal cancer and death from other malignancies or non-cancer causes stratified by endoscopic resection indication status (Figure 2). In both absolute and relative indication groups, the cumulative incidence of esophageal cancer-specific death remained 0% throughout the 5-year follow-up period, whereas the incidence of death from other malignancies and non-cancer causes increased steadily over time. Gray’s test was not performed for death from esophageal cancer because no such events occurred during the follow-up period.

**Figure 3:**
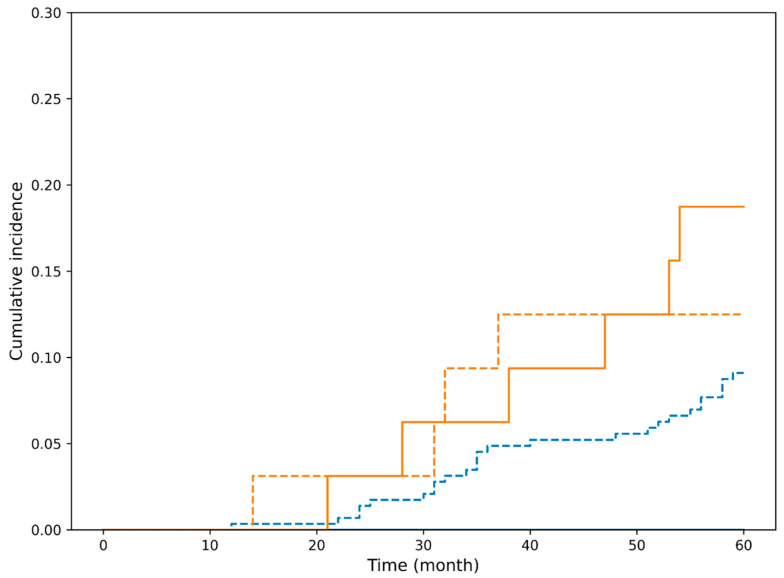
Cumulative incidence of esophageal cancer-specific mortality according to curative resection status in the overall cohort. Blue dashed line: The incidence of death from other malignancies and non-cancer causes in patients with curative resection. Orange dashed line: The incidence of death from other malignancies and non-cancer causes in patients with beyond indication. Orange solid line: The incidence of death from esophageal cancer in patients with beyond indication. Cumulative incidence functions for death from esophageal cancer (solid line, event of interest) and death from other malignancies and non-cancer causes (dashed lines, competing events) are shown, stratified by curative resection and beyond indication. The cumulative incidence of esophageal cancer-specific mortality differed significantly between the curative and beyond indication groups (Gray’s test, *p* < 0.001).

In contrast, in the non-curative resection group, the cumulative incidence of esophageal cancer-specific death increased early after treatment and plateaued thereafter, indicating a substantially higher risk of disease-specific mortality compared with the curative group (Figure 3). Cumulative incidence functions for death from esophageal cancer and death from other malignancies and non-cancer causes are shown, stratified by curative resection and beyond indication. The cumulative incidence of esophageal cancer-specific mortality differed significantly between the groups (Gray’s test, *p* < 0.001).

#### 3.3.3. Mortality Risk Factors in the Curative Resection Group

Univariable and multivariable Cox proportional hazards analyses were performed to identify risk factors for mortality in patients who achieved curative resection (Table 3). In the univariable analysis, lower body mass index (BMI) and older age were significantly associated with poorer overall survival.

In the multivariable model, lower BMI remained an independent predictor of mortality (hazard ratio [HR] per 1 kg/m^2^ decrease: 0.86, 95% CI: 0.73–0.98, *p* = 0.021), after adjustment for age and sex (Table 3). Furthermore, patients were classified into three BMI categories based on the World Health Organization criteria: low BMI (<18.5), normal BMI (18.5–24.9), and high BMI (≥25.0), and prognosis and BMI were also analyzed using categorical variables. Multivariate analysis demonstrated that the low BMI group had a significantly worse prognosis than the other two groups (Table 4).

These findings indicate that, among patients cured of superficial esophageal cancer, overall prognosis is strongly influenced by general health status and physiological reserve rather than by cancer-related factors.

#### 3.3.4. Association Between BMI and Mortality

**Figure 4:**
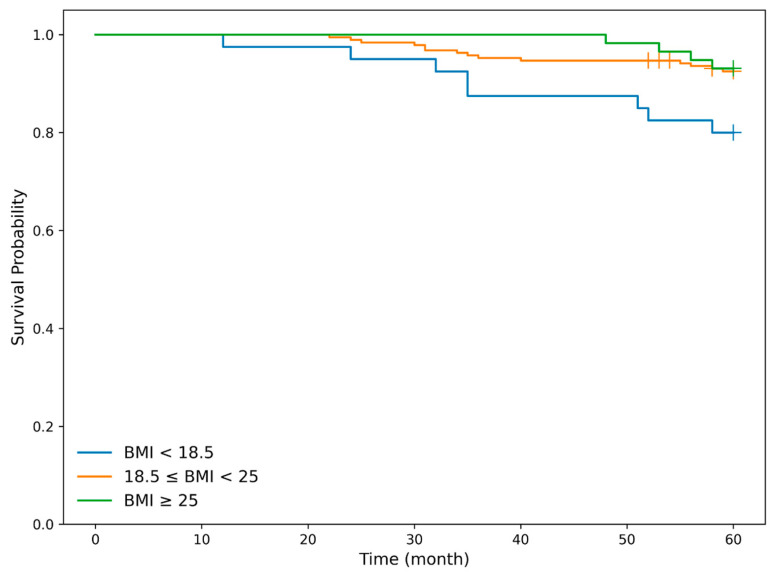
Kaplan–Meier curves for overall survival according to body mass index in the curative treatment cohort. Overall survival differed significantly among the BMI groups (log-rank test, *p* = 0.028). In pairwise comparisons, overall survival was significantly poorer in the low BMI (<18.5) group (blue solid line) compared with the normal BMI (18.5–24.9) group (orange solid line) (*p* = 0.013) and the high BMI (≥25.0) group (green solid line) (*p* = 0.043), whereas no significant difference was observed between the normal and high BMI groups (*p* = 0.852). Lower BMI was significantly associated with poorer OS in the curative resection group.

We next examined the association between body mass index (BMI) and mortality in the curative resection group, which constituted the primary endpoint population. Patients were classified into three BMI categories based on World Health Organization criteria: low BMI (<18.5), normal BMI (18.5–24.9), and high BMI (≥25.0). And overall survival (OS) was first evaluated using Kaplan–Meier analysis. Overall survival differed significantly among the BMI groups (log-rank test, *p* = 0.028). In pairwise comparisons, overall survival was significantly poorer in the low BMI group compared with the normal BMI group (*p* = 0.013) and the high BMI group (*p* = 0.043), whereas no significant difference was observed between the normal and high BMI groups (*p* = 0.852). Lower BMI was significantly associated with poorer OS in the curative resection group (Figure 4).

**Figure 5:**
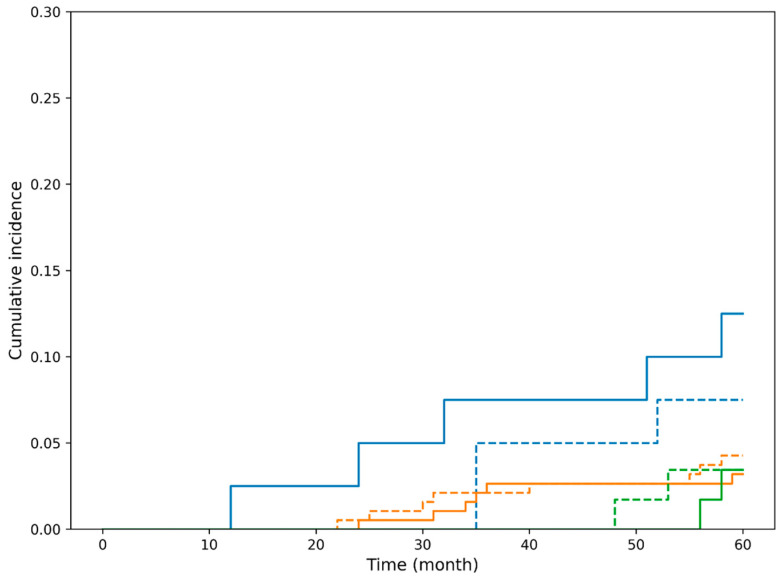
Cumulative incidence of non-cancer-related mortality according to body mass index in the curative treatment cohort. Cumulative incidence functions were estimated, and the association between BMI and non-cancer mortality was evaluated using the Fine–Gray subdistribution hazards model. Cumulative incidence functions are shown for death from non-cancer causes (solid line) and death from esophageal cancer or other malignancies (dashed line), stratified by BMI categories as stated above. The cumulative incidence of non-cancer-related death differed significantly among BMI groups (Gray’s test, *p* = 0.033). In pairwise comparisons, the cumulative incidence was significantly higher in the low BMI group (blue line) than in the normal BMI group (orange line) (*p* = 0.038), whereas no significant differences were observed between the low and high BMI groups (green line) (*p* = 0.250) or between the normal and high BMI groups (*p* = 1.000). Lower BMI was significantly associated with a higher cumulative incidence of non-cancer-related death.

Because no esophageal cancer-related deaths occurred in this group and mortality was driven by deaths from other causes, we further assessed the impact of BMI on non-esophageal cancer-related death using competing risk methodology. Cumulative incidence functions were estimated, and the association between BMI and non-cancer mortality was evaluated using the Fine–Gray subdistribution hazards model. Cumulative incidence functions are shown for death from non-cancer causes and death from esophageal cancer or other malignancies, stratified by BMI categories as stated above. The cumulative incidence of non-cancer-related death differed significantly among BMI groups (Gray’s test, *p* = 0.033). In pairwise comparisons, the cumulative incidence was significantly higher in the low BMI group than in the normal BMI group (*p* = 0.038), whereas no significant differences were observed between the low and high BMI groups (*p* = 0.250) or between the normal and high BMI groups (*p* = 1.000). Lower BMI was significantly associated with a higher cumulative incidence of non-cancer-related death (Figure 5).

These findings indicate that, among patients who achieved curative endoscopic resection, BMI functions primarily as a marker of overall physiological reserve and vulnerability to non-cancer mortality rather than as a determinant of esophageal cancer-specific prognosis.

### 3.4. Secondary Endpoint: Mortality Analyses in the Entire Cohort

We next extended the analysis to the entire cohort of 319 patients, including both curative and non-curative (beyond-indication) resections, to explore factors associated with overall and cause-specific mortality.

Because patients were at risk of dying from multiple mutually exclusive causes, competing risk analyses were first performed using the Fine–Gray subdistribution hazards model. In these models, treatment indication category (curative vs. non-curative) was strongly associated with the cumulative incidence of esophageal cancer-specific death (Figure 3). Patients with beyond-indication lesions had a markedly higher subdistribution hazard for esophageal cancer-related mortality than those who achieved curative resection.

**Figure 6:**
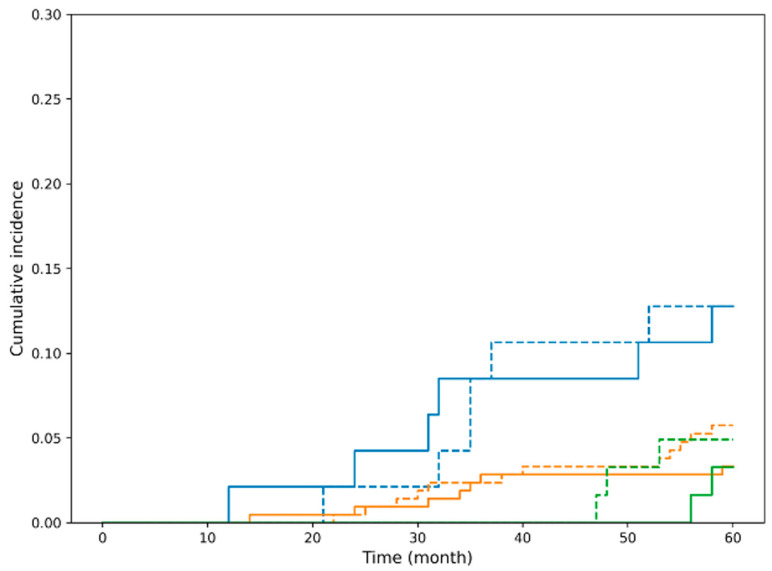
Cumulative incidence of non-cancer-related death according to body mass index in the overall cohort. Cumulative incidence functions are shown for death from non-cancer causes (solid lines; event of interest) and death from esophageal cancer or other malignancies (dashed lines; competing events), stratified by BMI categories based on World Health Organization criteria: low BMI (<18.5, blue line), normal BMI (18.5–24.9, orange line), and high BMI (≥25.0, green line). The cumulative incidence of non-cancer-related death differed significantly among BMI groups (Gray’s test, *p* = 0.017). In pairwise comparisons, the cumulative incidence was significantly higher in the low BMI group than in the normal BMI group (*p* = 0.021), whereas no significant differences were observed between the low and high BMI groups (*p* = 0.169) or between the normal and high BMI groups (*p* = 1.000).

By contrast, BMI was not associated with esophageal cancer-specific death. Instead, low BMI was significantly associated with a higher cumulative incidence of non-cancer-related mortality in the Fine–Gray model (Figure 6), indicating that host-related vulnerability rather than tumor biology drove long-term mortality in this cohort. Cumulative incidence functions are shown for death from non-cancer causes (solid lines; event of interest) and death from esophageal cancer or other malignancies (dashed lines; competing events), stratified by BMI categories based on World Health Organization criteria: low BMI (<18.5), normal BMI (18.5–24.9), and high BMI (≥25.0). The cumulative incidence of non-cancer-related death differed significantly among BMI groups (Gray’s test, *p* = 0.017). In pairwise comparisons, the cumulative incidence was significantly higher in the low BMI group than in the normal BMI group (*p* = 0.021), whereas no significant differences were observed between the low and high BMI groups (*p* = 0.169) or between the normal and high BMI groups (*p* = 1.000).

**Figure 7:**
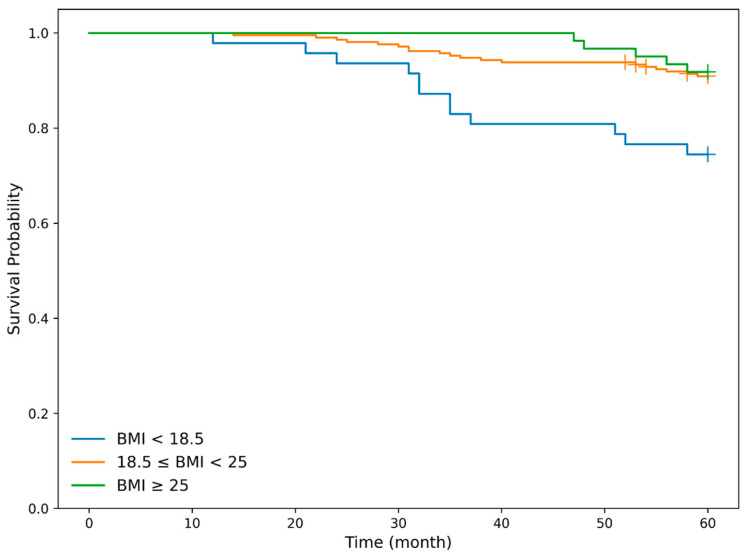
Kaplan–Meier curves for non-cancer-related mortality according to body mass index in overall cohort. The 5-year non-cancer-related survival rates were 89.1% in the low BMI group (blue line), 96.9% in the normal BMI group (orange line), and 97.6% in the high BMI group (green line). Kaplan–Meier analysis demonstrated a significant difference in non-cancer-related mortality among the three BMI groups (log-rank test, *p* = 0.026). Pairwise comparisons showed significantly poorer survival in the low BMI group compared with the normal BMI group (*p* = 0.011), whereas the difference between the low and high BMI groups did not reach statistical significance (*p* = 0.075). No significant difference was observed between the normal and high BMI groups (*p* = 0.952).

Kaplan–Meier survival curves were then generated to evaluate non-cancer-related survival. Patients with lower BMI had significantly worse non-cancer-related survival than those with higher BMI (log-rank test, *p* = 0.0026) (Figure 7). The 5-year non-cancer-related survival rates were 89.1% in the low BMI group, 96.9% in the normal BMI group, and 97.6% in the high BMI group. Kaplan–Meier analysis demonstrated a significant difference in non-cancer-related mortality among the three BMI groups (log-rank test, *p* = 0.026). Pairwise comparisons showed significantly poorer survival in the low BMI group compared with the normal BMI group (*p* = 0.011), whereas the difference between the low and high BMI groups did not reach statistical significance (*p* = 0.075). No significant difference was observed between the normal and high BMI groups (*p* = 0.952).

**Figure 8:**
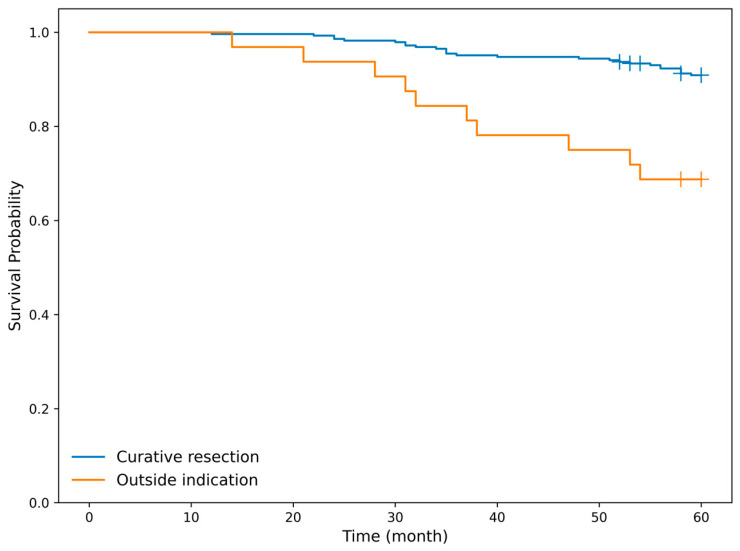
Kaplan–Meier curves for all-cause survival according to curative treatment status in overall cohort. All-cause survival differed significantly between the curative treatment group (blue line) and beyond indication group (orange line) (log-rank test, *p* < 0.001).

In addition, patients who underwent non-curative resection showed significantly poorer overall survival compared with those in the curative resection group (log-rank test, *p* < 0.001) (Figure 8).

Finally, univariable and multivariable Cox proportional hazards models were used to identify independent risk factors for all-cause mortality. In the full cohort, lower BMI was significantly associated with increased all-cause mortality in both univariable and multivariable analyses, whether BMI was treated as a continuous variable or categorized into clinically relevant groups. This association remained robust after adjustment for age and treatment indication category. Non-curative resection status was also an independent predictor of all-cause mortality (Table 5 and Table 6).

### 3.5. Recurrence Analysis and Additional Treatments for Patients with Non-Curative Endoscopic Resection

Metachronous and synchronous lesions were highly prevalent. Including cases identified outside the original study period (2020–2023), 175 metachronous or synchronous lesions (44%) were observed among 98 patients (31%) enrolled between 2008 and 2020. Statistical analyses revealed no significant associations between patient background factors and the occurrence of metachronous or synchronous lesions.

All non-curative resected patients were recommended additional therapy such as chemoradiotherapy or surgery. Among 32 patients with non-curative resection, 21 received additional prophylactic treatment and 11 did not because of age, comorbidities, or patient preference. Of those treated, 13 underwent CRT, 5 surgery, and 3 radiotherapy alone; 4 in the CRT group and 2 in the surgery group died of esophageal cancer. Among the 11 untreated patients, lymph node metastasis developed in 2; both achieved complete remission after additional therapy (CRT in one and heavy ion radiotherapy in the other) and are currently alive.

## 4. Discussion

In our previous retrospective cohort analysis, which was conducted internally as an exploratory study and has not been published, low BMI and older age were identified as potential risk factors for mortality within 5 years. These preliminary observations motivated us to continue patient enrollment and long-term follow-up to more rigorously evaluate this clinically important issue. In the present ambispective cohort, multivariate analysis confirmed that low BMI and advanced age were independent predictors of poor prognosis. Furthermore, competing risk analysis using the Fine–Gray model demonstrated that low BMI was specifically associated with an increased cumulative incidence of non-cancer-related death, whereas it had no significant impact on esophageal cancer-specific mortality.

Although obesity is often defined as BMI ≥ 25, BMI < 18.5 is increasingly recognized as the standard definition of underweight. Stratification into underweight, normal, and obese categories revealed that underweight status was a significant prognostic factor, particularly for non-cancer-related mortality. This distinction was clarified by the competing risk framework, which allowed us to disentangle host-related vulnerability from tumor-related lethality.

Among indication-eligible lesions, deaths due to other malignancies and non-cancer-related causes occurred at similar frequencies. Lifestyle risk factors such as alcohol consumption and smoking were prevalent across most patients, limiting their discriminatory value. In contrast, sarcopenia and frailty have recently been reported as adverse prognostic factors in various diseases, and our findings further support this concept. Our Fine–Gray results support the interpretation that BMI functions as a surrogate of frailty and physiological reserve rather than tumor aggressiveness.

BMI values were obtained at the time of endoscopic treatment. However, weight loss following treatment or additional therapies, especially surgery for other diseases or malignancies that subsequently occurred, was frequently observed. Such patients may require particularly careful follow-up. Although older age was also associated with worse prognosis, its hazard ratio was relatively modest (HR 1.09, 95% CI 1.03–1.15), suggesting that nutritional status may have a greater clinical impact than chronological age.

Prognosis differed significantly according to endoscopic treatment indication category, with beyond-indication lesions showing worse outcomes primarily due to disease-specific mortality. In the Fine–Gray model, treatment indication category was strongly associated with esophageal cancer-specific death, whereas BMI was not, indicating that tumor-related risk and host-related risk operate through distinct pathways. No significant difference was observed between absolute and relative indication lesions; however, this classification is based on pathological findings after endoscopic treatment. In real-world practice, lesions clinically suspected to be M3/SM1 may ultimately be reclassified as beyond-indication lesions due to lymphovascular invasion. Evaluating outcomes after additional treatment in such cases may be more clinically relevant.

Given the increasing use of diagnostic ESD for clinical SM (cT1b) lesions and the availability of multimodal salvage treatments such as CRT and photodynamic therapy, the number of beyond-indication lesions managed endoscopically may continue to increase. The three participating institutions are regional referral centers capable of radiotherapy, minimizing selection bias.

Despite excellent disease-specific outcomes, the 5-year survival rates for absolute and relative indication lesions (92% and 86%, respectively) were lower than those reported for endoscopically treated early-stage gastric or colorectal cancers. For example, consistent with national and multicenter cohort data, lesions meeting standard endoscopic indications for early gastric cancer—including differentiated pT1a and pT1b1 (<500 µm) without lymphovascular invasion—show excellent 5-year disease-specific survival (99.5%) and high overall survival (~93.9%) [15]. Similarly, early colorectal cancers with superficial submucosal invasion (<1000 µm) and negative lymphovascular invasion demonstrate favorable long-term outcomes with recurrence-free survival exceeding 97% [16]. This reason likely reflects the high prevalence of lifestyle-related risk factors and comorbidities among patients with esophageal cancer. Indeed, deaths due to head and neck cancer, gastric cancer, and other malignancies were common even among indication-eligible patients.

Importantly, this study was driven by the clinical concern that a substantial proportion of patients with superficial esophageal cancer, despite achieving curative endoscopic resection, die relatively early from causes unrelated to esophageal cancer. The present findings directly validate this concern by demonstrating that non-cancer-related mortality—particularly among underweight and frail patients—is a major determinant of long-term outcome.

These findings support our initial hypothesis that patients with superficial esophageal cancer curatively treated by endoscopic therapy frequently die from causes other than esophageal cancer at relatively early during follow-up. Previous studies have demonstrated that disease-specific survival after curative endoscopic submucosal dissection for superficial esophageal cancer is excellent, whereas overall survival is substantially influenced by deaths unrelated to the primary disease, including comorbid conditions and other malignancies [14].

Among patients with lesions treated within the endoscopic curative indication, approximately half of the deaths occurring within five years were attributable to causes other than the primary disease, with newly developed lung cancer and head and neck cancer accounting for a substantial proportion. Conversely, although not systematically analyzed, there were cases in which surveillance CT performed for lymph node assessment incidentally detected highly lethal malignancies such as lung or pancreatic cancer at an early stage, thereby enabling curative surgical treatment. These observations suggest that surveillance CT including the chest may be beneficial even for absolute indication patients with minimal risk of lymph node metastasis. Additionally, although annual endoscopic surveillance is recommended by current guidelines, shorter intervals may be warranted in high-risk patients.

In the present study, lower BMI was significantly associated with poorer other-cause survival, whereas no significant difference was observed between normal-weight and overweight/obese patients. These findings suggest that being underweight, rather than excess body weight, may be a more critical determinant of non-cancer-related mortality in this patient population. Several mechanisms may account for the unfavorable outcomes observed in underweight patients. Low BMI is often considered a surrogate marker of frailty, sarcopenia, and impaired nutritional status, all of which have been associated with increased vulnerability to comorbid conditions, infections, and treatment-related adverse events [17,18,19]. Additionally, underweight patients may have limited physiological reserve, rendering them more susceptible to non-cancer-related causes of death during long-term follow-up [20,21].

In contrast, overweight and obese patients did not exhibit inferior other-cause survival compared with those of normal weight. At first glance, this appears counterintuitive, as obesity is often considered a negative prognostic factor in oncology. However, this observation is consistent with the so-called “obesity paradox,” which has been reported in various oncologic and non-oncologic settings, wherein modestly elevated BMI is not necessarily associated with worse survival outcomes [22,23]. Although the underlying mechanisms remain unclear, preserved nutritional reserves and muscle mass may partially contribute to this phenomenon.

Taken together, these results highlight the clinical importance of baseline nutritional status in predicting long-term outcomes unrelated to cancer progression. Careful assessment and optimization of nutritional and functional status, particularly in underweight patients, may represent a potential strategy to reduce other-cause mortality and improve overall prognosis.

The strengths of this study include the relatively large cohort size and the long-term follow-up, which enabled comprehensive assessment of overall survival as well as cause-specific mortality. By analyzing disease-specific death, other cancer-related death, and non-cancer-related death separately, this study provides a detailed and clinically relevant evaluation of prognosis beyond conventional overall survival analysis. The combined evaluation of treatment indication and body mass index (BMI) offers novel insight into the prognostic impact of nutritional status in patients undergoing curative endoscopic treatment for superficial esophageal cancer.

Several limitations should also be acknowledged. This study included a retrospective cohort, which may have introduced selection bias and unmeasured confounding. BMI was assessed only at baseline and may not adequately capture longitudinal changes in nutritional status, body composition, or sarcopenia during follow-up. In addition, causal relationships between BMI, treatment indication, and survival outcomes cannot be established. The number of disease-specific death events was relatively small, which may have limited the statistical power for subgroup analyses. Finally, variations in clinical decision-making and advancements in endoscopic techniques over the study period may have influenced patient classification and outcomes.

BMI was assessed only at baseline and not modeled as a time-dependent covariate. Therefore, longitudinal changes in nutritional status, recurrence, or additional treatments were not incorporated into the risk models. This may have introduced some degree of misclassification. Nevertheless, baseline BMI remained a robust independent predictor of overall and non-cancer-related mortality, suggesting that it may serve as a simple and clinically practical surrogate marker of frailty and physiological reserve.

## 5. Conclusions

Despite excellent disease-specific survival after curative endoscopic resection for superficial esophageal cancer, overall survival was substantially affected by mortality unrelated to the primary disease. This adverse impact was particularly evident among patients with a low body mass index.

## Figures and Tables

**Table 1:** Baseline characteristics of patients with superficial esophageal cancer treated by endoscopic resection.

Endoscopic Treatment Indication		Curative Resection		Beyond Indication (*n* = 32)	Total (*N* = 319)
Characteristics		Absolute (*n* = 235)	Relative (*n* = 52)		
Age, years	Median (IQR)	70.0 (63.0–75.0)	71.0 (65.0–76.8)	75.0 (68.3–77.0)	70.0 (64.0–76.0)
Sex, *n* (%)	Male	210 (89%)	42 (81%)	29 (91%)	281 (88%)
	Female	25 (11%)	10 (19%)	3 (9%)	38 (12%)
BMI, kg/m^2^	Median (IQR)	22.3 (20.0–24.8)	21.0 (19.6–23.3)	22.1 (19.5–24.1)	22.0 (19.9–24.6)
BMI Category-Stratified WHO Classification, *n* (%)	Low: <18.5	34 (14%)	6 (12%)	7 (22%)	47 (15%)
	Normal: 18.5–24.9	151 (64%)	38 (73%)	22 (69%)	211 (66%)
	High: ≥25.0	50 (21%)	8 (15%)	3 (9%)	61 (19%)
Smoking status *n* (%)	Never	38 (16%)	10 (19%)	7 (22%)	55 (17%)
	Former	129 (55%)	18 (35%)	12 (38%)	159 (50%)
	Current	68 (29%)	22 (42%)	12 (38%)	102 (32%)
	Unknown	0 (0%)	2 (4%)	1 (3%)	3 (1%)
Alcohol consumption *n* (%)	Yes	193 (82%)	38 (73%)	26 (81%)	257 (81%)
	No	42 (18%)	11 (21%)	5 (16%)	58 (18%)
	Unknown	0 (0%)	3 (6%)	1 (3%)	4 (1%)
Tumor location *n* (%)	Ce	9 (4%)	3 (6%)	2 (6%)	14 (4%)
	Ut	35 (15%)	6 (12%)	6 (19%)	47 (15%)
	Mt	138 (59%)	29 (56%)	15 (47%)	182 (57%)
	Lt	43 (18%)	11 (21%)	7 (22%)	61 (19%)
	Ae	10 (4%)	3 (6%)	2 (6%)	15 (5%)
Histology *n* (%)	SCC	230 (98%)	50 (98%)	30 (94%)	310 (97%)
	Adenocarcinoma	5 (2%)	2 (2%)	2 (6%)	9 (3%)
Depth of invasion, *n* (%)	M1/M2	235 (100%)	0 (0%)	5 (16%)	240 (75%)
	M3	0 (0%)	37 (71%)	5 (16%)	42 (13%)
	SM1	0 (0%)	15 (29%)	6 (19%)	22 (7%)
	SM2	0 (0%)	0 (0%)	16 (50%)	16 (5%)
Lympho-vascular invasion, *n* (%)	Negative	235 (100%)	52 (100%)	19 (59%)	299 (94%)
	Positive	0 (0%)	0 (0%)	13 (41%)	20 (6%)
Lateral margin status (HM), *n* (%)	Negative	211 (90%)	45 (87%)	26 (81%)	282 (88%)
	Positive or X	24 (10%)	7 (13%)	6 (19%)	37 (12%)
Vertical margin status (VM), *n* (%)	Negative	235 (100%)	52 (100%)	28 (88%)	315 (99%)
	Positive or X	0 (0%)	0 (0%)	4 (13%)	4 (1%)
Metachronous or Synchronous lesions, *n* (%)	Yes	76 (32%)	15 (29%)	7 (22%)	98 (31%)
	No	159 (68%)	37 (71%)	25 (78%)	221 (69%)
Tumor size (mm)	Median (IQR)	20.0 (12.0–30.0)	22.0 (15.0–28.0)	23.0 (16.3–40.0)	20.0 (13.0–30.0)
Follow-up period (months)	Median (IQR)	92.0 (67.0–121.0)	88.0 (62.0–121.8)	79.0 (48.5–98.8)	89.0 (65.0–120.0)

**Table 2:** Five-year survival and causes of death according to endoscopic treatment indication (2008–2020).

	Curative Resection Cohort			Non-Curative	Total (*N* = 319)
	All (*n* = 287)	Absolute Indication (*n* = 235)	Relative Indication (*n* = 52)	Beyond Indication (*n* = 32)	
All-cause deaths within 5 years, *n* (%)	26 (9%)	18 (8%)	8 (15%)	10 (31%)	36 (11%)
Death from esophageal cancer, *n* (%)	0 (0%)	0 (0%)	0 (0%)	6 (19%)	6 (2%)
Death from other malignancies, *n* (%)	13 (50%)	10 (4%)	3 (6%)	2 (6%)	15 (5%)
Death from non-cancer causes, *n* (%)	13 (50%)	8 (3%)	5 (10%)	2 (6%)	15 (5%)
5-year overall survival rate	91%	92%	85%	69%	89%
5-year cause-specific survival rate	100%	100%	100%	81%	98%

**Table 3:** Univariable and multivariable Cox’s proportional hazards analyses for overall mortality in the curative treatment cohort.

Variables	Univariable Analysis		Multivariable Analysis	
	Hazard Ratio (95% C.I.)	*p*-Value	Hazard Ratio (95% C.I.)	*p*-Value
Age (per 1-year increase)	1.08 (1.03–1.13)	0.003	1.09 (1.03–1.15)	0.002
BMI (per 1 kg/m^2^ increase)	0.86 (0.76–0.98)	0.021	0.86 (0.75–0.98)	0.021
Sex: Male vs. Female	1.70 (0.40–7.18)	0.473	2.65 (0.60–11.70)	0.199
ER: Relative vs. Absolute	2.13 (0.93–4.89)	0.076	2.04 (0.87–4.75)	0.100
Metachronous or synchronous lesions: Yes vs. No	1.37 (0.62–3.03)	0.430		

CI, confidence interval; BMI, body mass index; ER, endoscopic resection indication. Multivariable models were adjusted for age, sex, and BMI category.

**Table 4:** Univariable and multivariable Cox’s proportional hazards analyses for overall mortality in the curative treatment cohort (BMI category).

Variables	Univariable Analysis		Multivariable Analysis	
	Hazard Ratio (95% C.I.)	*p*-Value	Hazard Ratio (95% C.I.)	*p*-Value
Age (per 1-year increase)	1.08 (1.03–1.13)	0.003	1.09 (1.03–1.15)	0.002
BMI: <18.5 vs. 18.5–24.9	0.35 (0.15–0.83)	0.017	0.30 (0.13–0.72)	0.007
BMI: <18.5 vs. ≥25.0	0.31 (0.09–1.04)	0.058	0.30 (0.09–0.99)	0.049
Sex: Male vs. Female	1.70 (0.40–7.18)	0.473	2.48 (0.56–10.99)	0.231
ER: Relative vs. Absolute	2.13 (0.93–4.89)	0.076	2.28 (0.98–5.29)	0.056
Metachronous or synchronous lesions: Yes vs. No	1.37 (0.62–3.03)	0.430		

**Table 5:** Univariable and multivariable Cox’s proportional hazards analyses for all-cause mortality in overall cohort.

Variables	Univariable Analysis		Multivariable Analysis	
	Hazard Ratio (95% C.I.)	*p*-Value	Hazard Ratio (95% C.I.)	*p*-Value
Age (per 1-year increase)	1.07 (1.02–1.12)	0.002	1.07 (1.02–1.12)	0.005
BMI (per 1 kg/m^2^ increase)	0.85 (0.76–0.94)	0.002	0.85 (0.76–0.94)	0.002
Sex: Male vs. Female	2.39 (0.57–9.93)	0.232	2.68 (0.64–11.21)	0.177
ER: Curative treatment vs. Beyond indication	4.01 (1.93–8.33)	<0.001	3.06 (1.46–6.44)	0.003
Metachronous or synchronous lesions: Yes vs. No	1.14 (0.57–2.29)	0.704	—	—

CI, confidence interval; BMI, body mass index; ER, endoscopic resection indication. Multivariable models were adjusted for age, sex, and BMI category.

**Table 6:** Univariable and multivariable Cox’s proportional hazards analyses for all-cause mortality in overall cohort (BMI category).

Variables	Univariable Analysis		Multivariable Analysis	
	Hazard Ratio (95% C.I.)	*p*-Value	Hazard Ratio (95% C.I.)	*p*-Value
Age (per 1-year increase)	1.07 (1.02–1.12)	0.002	1.07 (1.02–1.11)	0.005
BMI: <18.5 vs. 18.5–24.9	0.32 (0.15–0.66)	0.002	0.33 (0.16–0.68)	0.003
BMI: <18.5 vs. ≥25.0	0.28 (0.10–0.79)	0.017	0.33 (0.11–0.95)	0.039
Sex: Male vs. Female	2.39 (0.57–9.93)	0.232	2.45 (0.58–10.27)	0.221
ER: Curative treatment vs. Beyond indication	4.01 (1.93–8.33)	<0.001	2.98 (1.40–6.32)	0.005
Metachronous or synchronous lesions: Yes vs. No	1.14 (0.57–2.29)	0.704	—	—

## Data Availability

The data supporting the findings of this study are not publicly available due to ethical and privacy restrictions, as they contain information that could compromise the confidentiality of research participants. However, the data are available from the corresponding author upon reasonable request and with permission of the relevant institutional review boards.

## Abbreviations

The following abbreviations are used in this manuscript:

BMI	Body Mass Index
M	Mucosa
SM	Submucosa
MM	Muscularis Mucosae
IEE	Image-Enhanced Endoscopy
EMR	Endoscopic Mucosal Resection
ESD	Endoscopic Submucosal Dissection
CRT	Chemoradiotherapy
JCOG	Japan Clinical Oncology Group
HR	Hazard Ratio
CI	Confidence Interval
CT	Computed Tomography
CSS	Cause-Specific Survival
